# Machine vision-based detection method for buzzer iron cores

**DOI:** 10.1371/journal.pone.0354351

**Published:** 2026-08-11

**Authors:** Xiaoyang Liu, Chenxin Sun, Cheng Wang, Xupeng Huang, Rongjin Zhu, Chongyang Hu

**Affiliations:** Faculty of Automation, Huai'an University, Huaian, Jiangsu province, China; UNICAMP, University of Campinas, BRAZIL

## Abstract

As a fundamental acoustic component, the buzzer is widely used in various electronic systems. The iron core is a critical element in buzzers for supporting the coil, and it is currently fed primarily by mechanical methods. To further improve the automatic feeding efficiency of iron cores, a machine vision-based detection method is proposed to achieve core localization, pose recognition, and notch-angle measurement. The method first employs the Hough transform to locate iron cores on the vibratory tray and exclude overlapping cores. It then statistically counts the edge pixels around each core's center to select only those facing upward. Finally, by traversing the core's circumference, it pinpoints the notch localization and computes its angle. This providing the necessary data support for the automatic grasping and placement of iron cores by the manipulator. Experimental results demonstrate that the Hough transform algorithm adopted in this paper achieves a mean relative localization error of only 2.61%, a recognition precision of 100% for front-up iron cores, and an average notch-angle measurement deviation of 1.06°. Compared with YOLOv8, the proposed method offers clear advantages in both detection accuracy and practicality.

## 1. Introduction

Buzzers are essential acoustic components used in communication equipment, consumer electronics, and industrial control systems. They have a broad application spectrum and a substantial production scale, with an annual output exceeding 10 billion units [1,2]. As fundamental electronic components, buzzers face minimal technical barriers during manufacturing, and their production is mainly undertaken by small enterprises and family workshops [3]. At present, buzzer manufacturing relies chiefly on manual labor assisted by small-scale automated equipment [4], and this specific industrial structure limits the further improvement of automation levels.

The iron core is a key supporting component in the electromagnetic drive structure of a buzzer, primarily serving to provide a base for coil winding and strengthen the magnetic field [5]. Its feeding efficiency is critical to automated buzzer production. At present, spiral vibratory trays are mainly used for orienting, sorting, and automatically feeding the cores [6]. Such mechanical feeding methods demand high machining accuracy of the parts and are prone to jamming. Replacing them with a robotic manipulator that can accurately pick and place the cores would markedly improve feeding efficiency [7]. Therefore, this study proposes a machine vision-based detection method for buzzer iron cores, which outputs the localization, pose and notch-angle information required for the robotic manipulator to complete picking and placing operations.

Spurred by recent advances in automation, intelligent technologies and Industry 4.0 trends, machine vision technology, which is valued for its high accuracy, efficiency, and reliability, has become indispensable in modern industrial production. It is now widely applied in critical tasks such as localization, identification, and measurement [8–10]. Machine vision improves production efficiency and reduces reliance on manual labor. Moreover, machine vision enables real-time process monitoring and quality control through precise image analysis and processing, thus improving product quality and enhancing process consistency. Furthermore, its strong adaptability and flexibility under complex working conditions allow it to address various industrial challenges, delivering solid technical support for the automation and intelligent upgrading of manufacturing lines [11–13].

In this study, the detection of buzzer iron cores is a highly integrated machine vision task that simultaneously demands accurate localization, pose recognition, and angular measurement. Although little published research specifically addresses machine vision detection of buzzer iron cores, extensive studies on related applications serve as theoretical and practical references. These studies cover industrial part measurement, robot-guided visual localization, and product classification on automated production lines. The algorithms, image-processing techniques, and error-analysis methods developed in these studies provide a valuable reference. They underpin the stable localization, reliable pose recognition, and accurate notch-angle measurement required in the detection of buzzer iron cores.

The localization function in machine vision aims to determine the position or orientation of an object in the image. Typical techniques include edge detection, template matching. For instance, Jinjiang Wang et al. [14] employed the Hough transform algorithm to extract the region of interest (ROI). This operation precisely localized the wine bottle and eliminated irrelevant background, thereby achieving a defect-detection accuracy of 99.6%. Similarly, Qinbang Zhou, Renwen Chen et al. [15] fused multi-scale Hessian matrices to screen potential regions from car-body images, enabling accurate defect localization. Their automated detection system attained 95.6% accuracy for dent defects and 97.1% accuracy for scratches.

The recognition function in machine vision technology is to identify and classify objects or features within an image. The system first analyzes and processes the image to extract features such as shape, color, and texture. It then compares these features with stored models or standards to determine the object's identity or category. For instance, Te-Hsiu Sun et al. [16] employed median filtering, threshold segmentation, and morphological operations to enhance the visibility of surface defects in images. Through subsequent feature extraction, they successfully classified defects on electrical-contact surfaces. They achieved 100% accuracy for detecting excess metal and side cracks, and 96.7% accuracy for edge fractures and back cracks. Durga Prasad Penumuru et al. [17] proposed a generic approach for automatic material recognition that integrates machine vision and machine learning. They extracted the red, green, and blue components of the RGB color model from the material under test. After training machine-learning models with these color features, they realized automatic identification of aluminum, copper, medium-density fiberboard (MDF), and mild steel. In a related task, M. M. Sofu [18] employed the K-means algorithm to segment apples from the background in binary images. The fruits were then classified by color, size, and weight, attaining 96% classification accuracy.

The measurement function refers to the use of machine vision technology to precisely measure dimensions, distance, or other physical properties of an object. Before conducting measurements, the system is usually calibrated so that results in pixels can be converted into real-world units. For example, using an edge-detection algorithm, Desmond K. Moru [19] extracted the outer contour of a gear and refined it in Vision2D software to remove spurious edge pixels. This refinement achieved sub-pixel accuracy with a maximum measurement error of only 0.004 mm. Yuanyuan Tian [20] employed a least-squares sub-pixel edge detector to locate edges and corners of mechanical parts, realizing a non-contact vision scheme with measurement errors limited to 0.006 mm. Yanli Yang [21] processed belt images with a fast segmentation algorithm, enabling online detection of longitudinal tears and lateral deviations directly from binary images of the conveyor belt.

Based on technical approaches, machine vision methods can be categorized into three types: traditional image-processing-based methods, including edge detection, threshold segmentation, and region growing; machine learning-based methods such as Support Vector Machine (SVM), Random Forest (RF), and K-Nearest Neighbors (KNN); and deep learning-based methods like Convolutional Neural Networks (CNN), YOLO, and SSD [22–25]. In the studies cited above, traditional image-processing and machine-learning approaches are predominantly employed. To further boost the performance of machine vision in industrial automation, researchers are now exploring new algorithms and technologies, especially deep learning. These advances aim to overcome the limitations of conventional approaches and expand the use of vision systems on the factory floor [26]. In both localization and recognition stages, deep learning algorithms such as YOLO enable real-time object detection. For instance, Jing Zhang et al. [27] proposed an industrial-part detection method based on an improved YOLOv3. B The authors re-clustered anchor parameters via K-means and adopted a multi-scale strategy to tailor the network for tiny industrial workpieces, addressing the drawbacks of conventional approaches. Compared with the original YOLOv3, the enhanced model improves accuracy by 1.52 percentage points and cuts inference time by 7.25ms. Tong Liu [28] modified the original YOLOv3 architecture: Darknet53 is kept as the backbone, coordinate-attention modules are inserted inside its residual blocks, and the Mish activation function is adopted. In addition, a PnP algorithm is employed to compute the 6-DoF pose of the target part. These enhancements give the network high accuracy and strong robustness for 3-D pose estimation of industrial components, offering an effective solution for related applications.

However, the end-to-end advantage of deep-learning algorithms is not applicable to the detection project of buzzer iron cores. The project is inherently multi-task-oriented. It demands core localization, pose recognition, and notch-angle measurement, each with distinct requirements that a single deep model can hardly satisfy [29,30]. Meanwhile, deep-learning models entail high computational costs. Since the project requires 10-megapixel resolution images to guarantee accuracy, adopting such models would significantly increase the computational load. In summary, to simultaneously guarantee accuracy and efficiency, this work adopts traditional image-processing techniques rather than deep learning methods.

This paper innovatively proposes a dedicated detection method for buzzer iron cores. Exploiting the fact that a buzzer iron core is circular, the method first employs the Hough transform algorithm to locate every core in the vibratory tray and eliminates overlapping cores by checking inter-center distances. Next, it extracts edges in the central region of each core and distinguishes front-up from back-up cores by counting edge pixels. Finally, it traverses the perimeter to find the notch and then computes the notch angle of the core. These detection data are used to guide the robot to pick up only those cores that are front-up while rotating each one so that its notch is delivered at the desired angle.

The main contributions of this work are:

1) An innovative detection method for buzzer iron cores: A machine vision-based detection method for buzzer iron cores is proposed for the first time, integrating modules for core localization, pose recognition, and angle measurement. Through the synergy of machine vision and image recognition technologies, the method can be deployed on automated iron core feeding equipment and significantly improves feeding accuracy and efficiency.
2) High-precision localization: This study employs the Hough transform algorithm, yielding an average relative localization error of only 2.61%. By computing the distance between circle centers, overlapping cores are rejected, so that only the topmost core in an overlap is located. In addition, cores in a tilted posture appear elliptical and are therefore missed by the Hough transform. Thus, the algorithm naturally filters out objects that are hard to grip, while ensuring accurate core localization.
3) Accurate pose recognition: This study exploits the fact that back-up cores have a protruding central pillar. By counting edge pixels in the neighborhood of the detected circle center, it achieves 100% accuracy in distinguishing front-up from back-up cores.
4) Reliable angle measurement: This study achieves accurate detection of the iron core notch by circumferentially traversing and verifying pixel continuity. The system then measures the notch angle by referencing the core's center coordinates, yielding an average angular error of 1.06°.

## 2. Methods & materials

### 2.1. Image acquisition

The buzzer iron core detected in this study is an Fe-Ni alloy with a silver-white metallic luster. It is a material commonly employed for buzzer iron cores. It consists of a circular iron disk joined to a central cylindrical pillar. There is a U-shaped notch on the disk edge to facilitate the passage of the copper wire of the electromagnetic coil.

**Fig 1:**
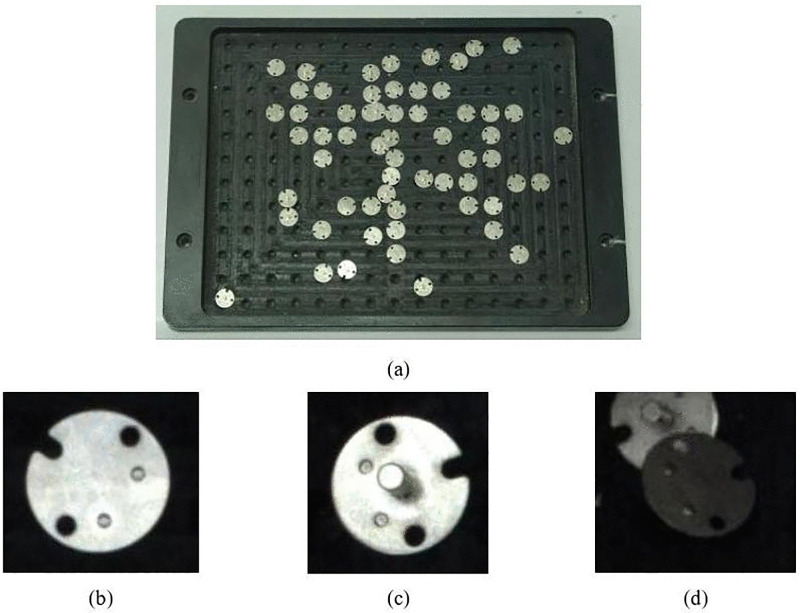
Images of iron cores. (a) The iron cores distributed within the vibratory tray (b) The front-up iron core (c) The back-up iron core (d) The titled iron core.

To facilitate the detection of the iron core's localization and its pickup by the robotic manipulator, this study designs a vibratory tray whose surface is perforated, inspired by tray used for screws and similar parts. When the tray vibrates, the cores spread out in a fairly even layer as shown in Fig 1(a). Three postures are observed: the pillar of some cores drops into a hole, leaving the smooth front face upward; the pillar points up, i.e., the reverse side faces upward; with low probability, a core tilts sideways when its pillar fails to enter any hole. Figs 1(b)–(d) illustrate these three orientations after vibration.

**Fig 2:**
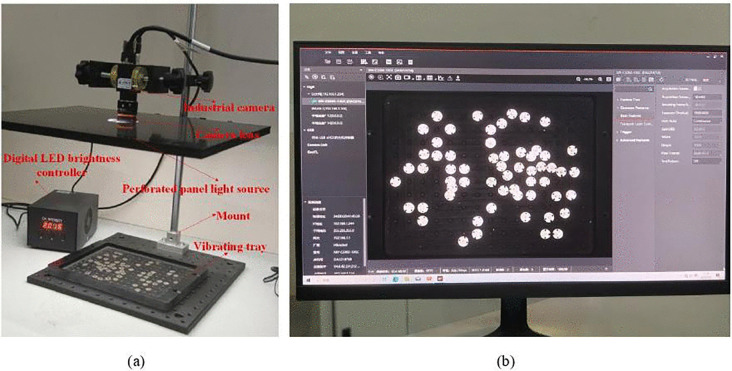
Image-acquisition module. (a) Image acquisition environment (b) Vision Master UI.

To capture clear images of the iron cores, the experiment built a complete image-acquisition system that simulates a real industrial environment, as shown in Fig 2(a). The system consists of an industrial camera, lens, illumination unit, brightness controller, mount, and vibratory tray. In this study, the camera employed is the HIKROBOT industrial model MV-CS060–10GC, paired with an MVL-HF0828M-6MPE lens whose focal length is 8 mm. The image-acquisition system is assembled as follows. First, place the vibratory tray containing the iron cores in the center of the support base. Then mount the industrial camera on the sliding guide rail of the shooting bracket, positioning the camera directly above the geometric center of the vibratory tray with a top-down overhead view to minimize lens distortion. Finally, set the shooting height of the lens to 37.5 cm and the field of view to 25 cm × 16 cm, ensuring the entire vibratory tray is fully contained within the captured image. To eliminate interference from ambient and indoor lighting, a 300 mm × 400 mm perforated surface light is fixed at a height of 40 cm. A digital LED brightness controller is used to adjust the illumination so that every region inside the tray is evenly lit. Finally, the image-acquisition module is adjusted in *Vision Master*, the bundled software of the HIKROBOT, as shown in Fig 2(b): image resolution is set to 3072 × 2048, exposure time to 5500 µs, and gain is enabled. One image set is acquired per vibration cycle of the tray.

White light is used for all experiments in this study. Its balanced spectrum clearly reveals subtle surface textures of iron cores and maximizes target-background contrast. Since our algorithm relies entirely on grayscale images, monochromatic light would distort grayscale distribution and disrupt subsequent processing. Hence, white light is chosen as the illumination source.

### 2.2. Overall scheme

**Fig 3:**
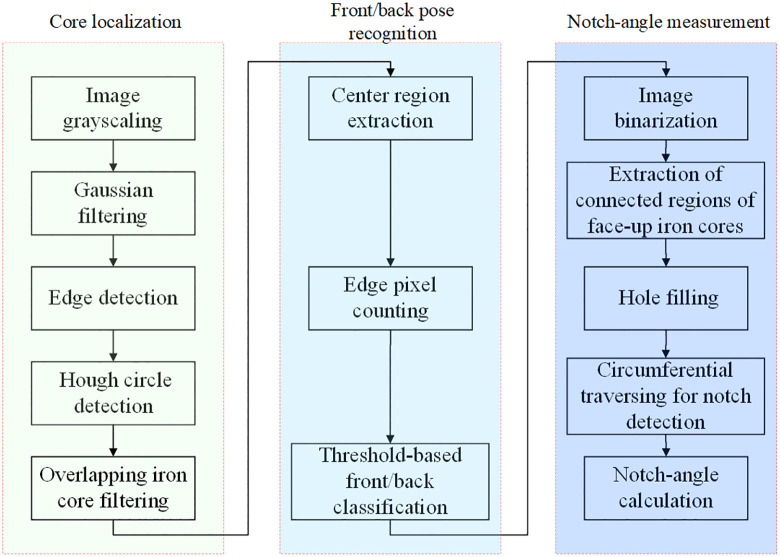
Detection procedures of iron cores.

To enable automated feeding of the iron core, this study proposes the processing steps shown in Fig 3. As can be seen in Fig 3, the complete iron core detection consists of 12 steps divided into three main stages: core localization, front/back pose recognition, and notch-angle measurement.

1) Core localization: Core localization is primarily used to guide the robotic manipulator in picking cores from the tray. In this study, the Hough transform algorithm is employed to detect and locate the circular cores in both front-up and back-up orientations. Cores that overlap are filtered out by checking the distance between circle centers, since overlap prevents reliable suction. Cores in a tilted pose appear elliptical and cannot be picked up, so their positions are ignored.
2) Front/back pose recognition: A back-up core has its pillar protruding, which blocks the pick-up by the robotic manipulator. The system is therefore expected to identify which cores are front-up and graspable. In the acquired images, the center region of a back-up core shows strong gray-level variations caused by the pillar, whereas a front-up core has a smooth, uniform center. This difference produces more edge pixels in the pillar area after edge detection. Therefore, counting the number of edge pixels within the core's central region enables reliable front/back pose identification.
3) Notch-angle measurement: To facilitate coil winding, the robotic manipulator must rotate each core so that its notch faces a predetermined orientation. To compute the notch angle, the input image is first binarized and the connected region of each front-up core is extracted. Internal holes are then filled to prevent the two circular holes from being mis-detected as the notch. Next, the system traverses the perimeter to locate the notch. Finally, the notch angle is calculated from the core's center coordinates.

### 2.3. Core localization

Since cores appear circular in the images regardless of their orientation, this study employs the Hough transform for circle detection and localization. To ensure the efficiency and accuracy of the Hough transform, the images must undergo grayscale conversion, Gaussian filtering, and edge detection. Circle detection based on the Hough transform relies solely on edge information, making color irrelevant. Thus, the RGB image is converted to a single-channel grayscale via weighted averaging, thereby discarding color information and reducing data dimensionality. Noise inevitably enters during imaging and transmission, impairing edge quality, so Gaussian filtering is applied to suppress it. Finally, the Canny operator extracts the image edges.

**Fig 4:**
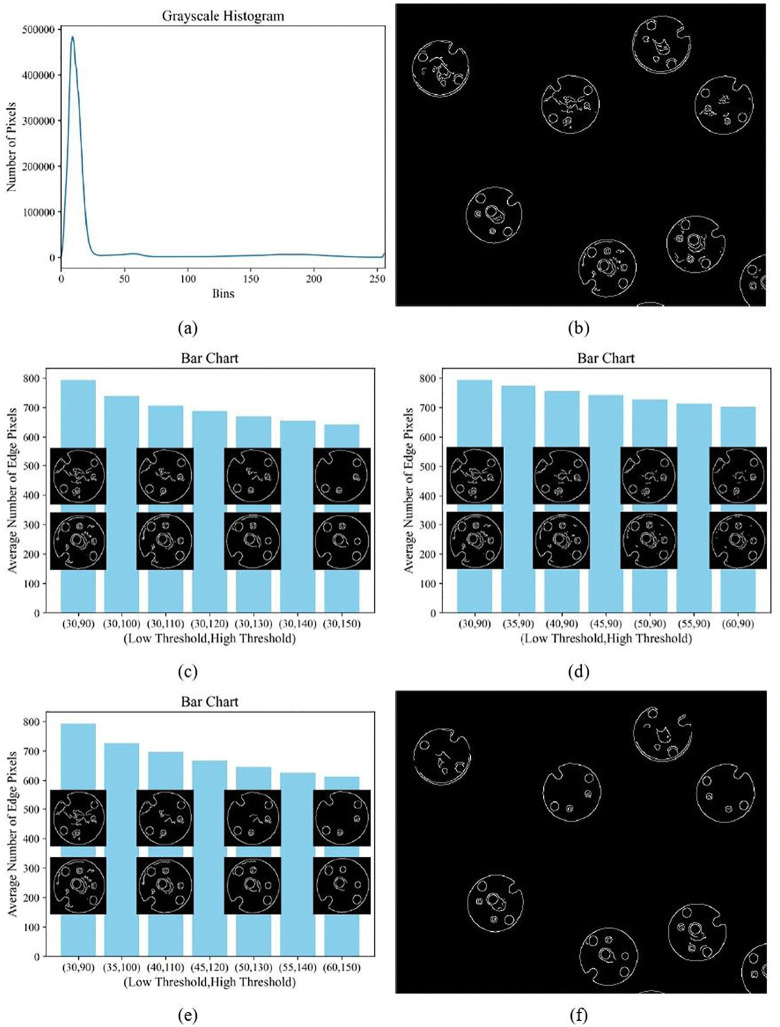
Edge detection module images. (a) Gray-level histogram (b) Initial edge map (c) Plot of the variation in the average pixel count across 20 images as the upper threshold is raised (d) Plot of the variation in the average pixel count across 20 images as the lower threshold is raised (e) Plot of the variation in the average pixel count across 20 images when both upper and lower thresholds are increased simultaneously (f) Final edge map.

The grayscale histogram of the image, shown in Fig 4(a), is examined prior to applying the Canny operator to characterize the gradient distribution. In the grayscale histogram, most pixels are concentrated in the low-gray region, corresponding to the vibratory tray background. To prevent the background from being misidentified as edges, the lower threshold can be initially set just after the first pronounced dip in the grayscale histogram, approximately 30. The upper threshold is initially set to three times this value, 90. This threshold pair yield the initial edge detection result in Fig 4(b). Visually, the detected core edges match the real image and are continuous, indicating that the strong-edge detection is basically correct. However, small spurious edges appear inside some cores. This is probably because the lower threshold is slightly too low and weak edges contain noise, or because the upper threshold is still too low and some noise or weak edges that should have been suppressed are erroneously accepted as strong edges.

To identify the cause of the spurious edges inside the cores and to determine the optimal upper and lower thresholds, 20 single-core (an equal number of cores are front-up and back-up) images randomly distributed in the vibratory tray are selected for Canny edge-detection tests. Using the control-variable method, the upper threshold is increased in steps of 10 and the lower threshold in steps of 5, first individually and then simultaneously. For each threshold pair, the mean number of edge pixels across all images is computed and compared with the corresponding edge map. Figs 4(c)–(e) present these comparisons in a “bar chart + edge map” format, showing how the mean pixel count varies when only the upper threshold, only the lower threshold, or both thresholds are raised.

As shown in the figure, whether the lower and upper thresholds are increased individually or simultaneously, the average number of edge pixels across the 20 images decreases and the spurious responses in the edge map are diminished. This indicates that setting either threshold too low introduces edge noise. Based on this principle, the lower and upper thresholds are simultaneously raised from their initial values while the edge-map is monitored, and the adjustment continues until edge noise is almost completely eliminated across all images, subject to the constraint that genuine edge loss is kept to a minimum. After iterative comparisons, the optimal pair is found to be 55 for the lower threshold and 140 for the upper threshold. With these settings, the final edge detection result of iron cores is displayed in Fig 4(f). As can be seen from the figure, although a few minor edge details are missing, the detected outline still matches the actual cores closely and remains sufficiently clear for subsequent Hough transform localization. Moreover, the interior of the cores is virtually free of noise, greatly facilitating the next step of front/back pose recognition.

The Hough transform is a shape-detection algorithm that can be used for circle detection and is widely employed in computer vision and image-processing. Since the iron core detected in this study is circular, the Hough transform can be adopted for its localization. The algorithm maps every edge pixel into a three-dimensional parameter space, where local maxima correspond to candidate circle centers and radii, thus enabling robust circular-object detection.

**Fig 5:**
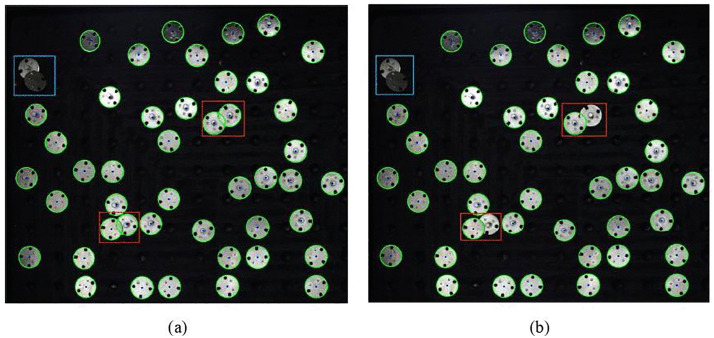
Circle detection images. (a) Preliminary circle detection results (b) Final circle detection results.

The iron core in this study has a radius of approximately 60 pixels. Considering errors caused by distortion, perspective and other factors, as well as iron cores with minor deviations between geometric dimensions and manufacturing tolerances, the radius search range in the parameter space is set from 57 to 63 pixels. Meanwhile, when iron cores overlap with one another, the lower cores are blocked and cannot be picked up by the robotic manipulator. Therefore, after circle detection, it is necessary to eliminate the circles corresponding to occluded iron cores by calculating the distance between circle centers. The detailed steps are as follows: if the distance between two centers is smaller than the sum of their radii, the two circles are regarded as overlapping ones. The circle with fewer edge pixels will be removed. In this way, only the topmost iron core among overlapping objects can be retained in the detection results. Fig 5(a) and Fig 5(b) show the detection results before and after this suppression. In Fig 5(a), the Hough transform accurately localizes all front-up or back-up cores, including those in the overlap region (red box), except for the tilted core (blue box). Fig 5(b) demonstrates that the suppressed image contains only the topmost core in each overlap, with the occluded ones removed.

### 2.4. Front/back pose recognition

Owing to the excessively protruding cylindrical pillar on the back-up iron core, the robotic manipulator is unable to pick it up. Consequently, after localization, the core's orientation must still be identified. Comparison between the two orientations reveals that the center region of a front-up iron core is relatively smooth, with negligible grayscale variation. In contrast, the back-up iron core exhibits significant grayscale changes in the center due to the presence of the cylindrical pillar. In edge maps, more pronounced grayscale changes correspond to a greater number of edge pixels. Thus, the orientation of the iron core can be determined by counting edge pixels within the central region.

**Fig 6:**
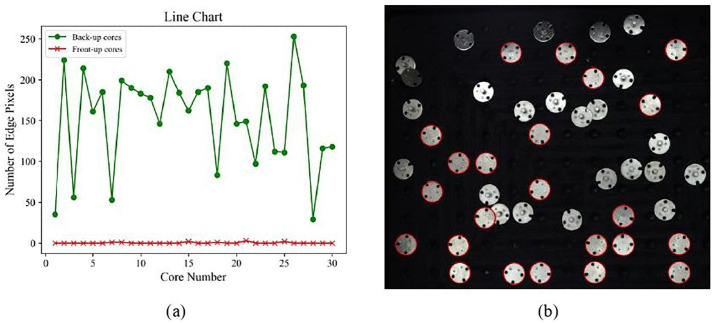
Front/back pose recognition module images. (a) Distribution of edge-pixel counts in the central region for front-up and back-up iron cores (b) Pose recognition result visualization.

First, the center of the iron core is taken as the center of the circle detected by Hough transform, and the central region is extracted from the edge image via AND operation. The number of edge pixels within this central region is then counted, and an appropriate threshold is set to distinguish front-up from back-up cores. The appearance of a core may differ slightly when it lies in the center of the image or near the border. Therefore, 30 front-up and 30 back-up cores are evenly distributed on the vibration tray to determine a reliable threshold. The distributions of edge-pixel count in their central regions are plotted in Fig 6(a). The red curve denotes front-up cores (mean = 0.33, variance = 0.593); the green curve denotes back-up cores (mean = 176.5, variance = 3421.025). Evidently, front-up cores show edge-pixel counts mostly at zero with little variation, whereas back-up cores exhibit high counts and large fluctuations. Given that only front-up cores can be grasped by the robotic manipulator, the task demands high precision: missed detections of front-up cores are tolerable, whereas false positives are unacceptable. Therefore, the maximum value of 3 for front-up cores is taken as the threshold. If the central-region pixel count is below 3, the core is classified as front-up; otherwise, it is back-up. Fig 6(b) shows the resulting pose labels: all the labeled cores are front-up.

### 2.5. Notch-angle measurement

**Fig 7:**
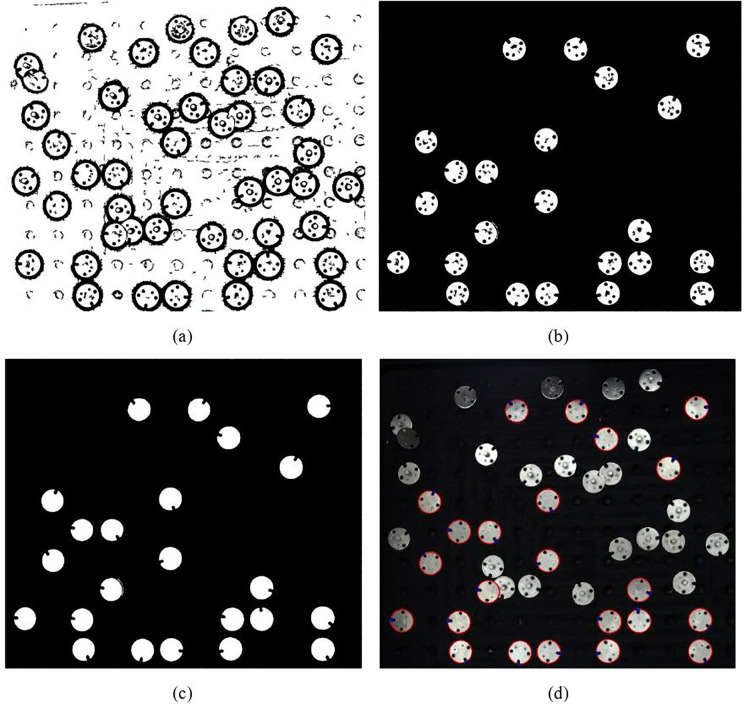
Notch detection module images. (a) Binarized image (b) Extraction result of front-up iron cores (c) Hole-filling result (d) Notch localization visualization.


After a front-up iron core is picked up by robotic manipulator, its notch must be aligned to a fixed angular position. Hence, the notch angle has to be measured. Because the core is circular, the notch disrupts its otherwise continuous edge contour. Leveraging this feature, the system traverse the perimeter to locate the notch, and the notch angle is then calculated from the center coordinates. The detailed procedures are as follows:

1) Image binarization: The image is binarized using an adaptive-threshold segmentation algorithm, and morphological operations are applied to suppress noise, as shown in Fig 7(a). After binarization, most of the core—having high gray values—is set to white, while the notch, the circular holes, and parts of the lug—having low gray values—are set to black.
2) Extraction of connected regions of front-up iron cores: Based on the front-up screening results, front-up cores are extracted from the binary image by AND-operation with the black mask. As shown in Fig 7(b), all connected regions of front-up iron cores in the binary image are completely extracted, which effectively eliminates interference from other cores and background noise.
3) Hole filling: The circular holes in the iron core lie near the edge and would disturb the subsequent circumference traverse used for notch localization. Therefore, hole filling is performed to ensure accurate notch detection. The result is shown in Fig 7(c), where the two circular holes in the core have been filled.
4) Circumferential traversing for notch detection: Each pixel on the circumference is traversed. If its position is not within the iron core connected component, the pixel is deemed to lie inside the notch. The localization outcome is shown in Fig 7(d): red and blue dots are the circumferential pixels, red denoting points inside the iron-core component and blue denoting points inside the notch.
5) Notch angle calculation: As the detected notch contains several pixels, the centroid of these pixels is taken as the notch coordinates to maximize positional accuracy. Connect the notch position to the center of the iron core to form a line segment, then take horizontally rightward as the positive direction and use equation (1) to compute the angle between this line segment and the horizontal. The angle ranges from -π to π, which corresponds to −180° to 180°.

$$\begin{array}{c} \partial =arctan\frac{a-x}{b-y} \end{array}$$ (1)

In the above equation,∂ denotes the notch angle; (*a*, *b*) are the average pixel coordinates of the notch, and (*x*, *y*) are the center coordinates of the buzzer iron core.

## 3. Experiments and results

All experiments in this section are conducted on the Windows 10 operating system. Specifically, experiments 3.1, 3.2, and 3.3 are implemented using the OpenCV module, while experiment 3.4 utilize the PyTorch module. The experimental hardware configuration is detailed in Table 1.

**Table 1:** Environment and configuration requirements.

Parameters	Configuration
CPU	Intel Core i9- 10920X CPU@3.50GHz
GPU	NIVDIA GeForce RTX 3090 GPU
RAM	32GB
GPU memory size	24GB
Operating systems	Windows 10
Deep learning architecture	Pytorch1.10.0 + Cuda11.3
Virtual environment	Anaconda3
Data processing	Python3.11

### 3.1. Core localization accuracy experiment

**Fig 8:**
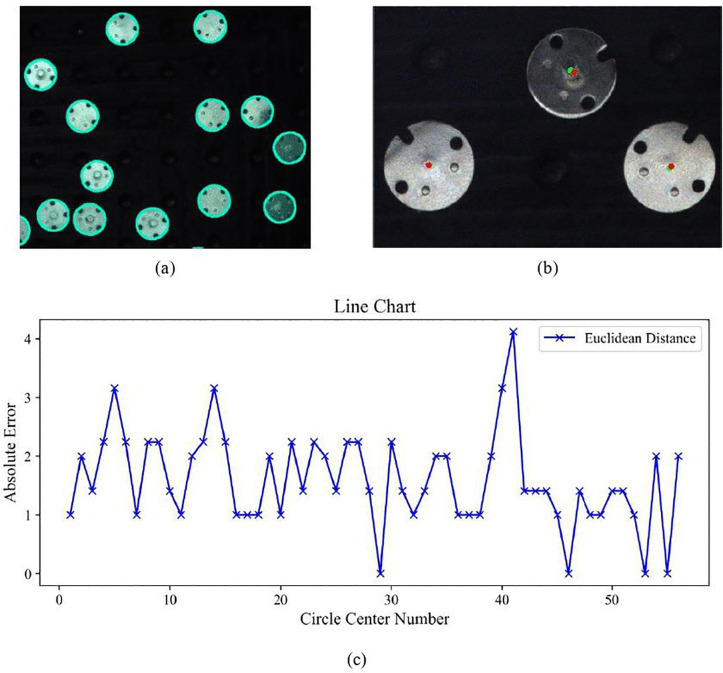
Core localization accuracy experimental images. (a) Pixel-level annotation visualization (b) Map of the localization deviation between the detected circle centers and the ground-truth centroids (c) Line chart of absolute localization error for core localization.

Relative to other stages, core localization is straightforward, yet its accuracy is crucial for guiding the robotic manipulator to pick up the core and for calculating the notch angle. Owing to lens distortion, optical perspective, and manufacturing tolerances of the cores, some cores do not appear as perfect circles in the images. Consequently, the center coordinates obtained by the Hough-circle detector may deviate from the ground-truth centroid. To evaluate the accuracy of the Hough-circle localization, a dataset of 200 cores (front-up or back-up) is prepared. Each core is annotated at the pixel level (including all holes and notches) using the software Supervise.ly, and an example is shown in Fig 8(a). The geometric centroid of every annotated region is taken as the ground-truth centroid. Simultaneously, the proposed Hough transform algorithm is applied to detect the center of each core. Fig 8(b) illustrates the localization errors for several cores: green dots mark the ground-truth centroids, and red dots mark the centers detected by Hough transform algorithm. The two sets are in close agreement. To better evaluate the localization accuracy of Hough transform algorithm, the Euclidean distance between the ground-truth centroid of each core and the detected center is calculated as the absolute localization error.

In the 2-D space of this study, let the ground-truth core centroid be P = (*x*₁, *y*₁) and the center detected by the Hough transform algorithm be Q = (*x*₂, *y*₂). The Euclidean distance between them is given by Equation (2):


$$\begin{array}{c} \text{Absolute Error}=\text{d(P,Q)}=\sqrt{\text{(}x_{\text{1}}-x_{\text{2}}{)}^{\text{2}}+(y_{\text{1}}-y_{\text{2}}{)}^{\text{2}}} \end{array}$$ (2)

The ratio of the absolute localization error to the true value is defined as the relative localization error. In this study, the equivalent radius r of each core is computed from its pixel-level annotated region and is taken as the true value. The formula for the relative error is given by Equation (3):


$$\begin{array}{c} \text{Relative Error}=\frac{\text{Absolute Error}}{\text{True Value}} \end{array}$$ (3)

Using the given formula, the average absolute localization error over 200 cores is found to be 1.57 pixels. Fig 8(c) displays a subset of these absolute error data. As shown in this figure, the error samples are uniformly distributed on both sides of the average error value, which indicates that the experimental dataset has no obvious offset and proves the reliability and stability of our measurement results. Furthermore, the figure also reveals that the Hough transform algorithm adopted in this paper produces small localization errors: most are below 2 pixels, and the maximum is only 4.12 pixels. The corresponding mean relative localization error is just 2.61%. Practical tests confirm that, within this error range, the robotic manipulator can still reliably pick up front-up cores.

To further verify the reliability and statistical validity of the localization error experimental data, a 95% confidence interval statistical analysis is performed on 200 sets of absolute localization error data of iron cores in this study.

The calculation formula for the 95% confidence interval of the population mean is given by Equation (4):


$$\begin{array}{c} CI=\overset{―}{x}\pm z_{\left\{\frac{\alpha }{\text{2}}\right\}}\cdot \frac{s}{\sqrt{n}} \end{array}$$ (4)

where $\overset{―}{x}$ denotes the sample mean of absolute localization error; $z_{\left\{\frac{\alpha }{\text{2}}\right\}}$ represents the two-tailed critical value of the standard normal distribution corresponding to the 95% confidence level ($z_{\text{0}.\text{0}\text{2}\text{5}}$=1.96); *s* is the sample standard deviation of localization error; *n* stands for the total number of experimental samples.

Using the given formula, the calculated 95% confidence interval of the iron core absolute localization error is [1.42, 1.72] pixels. The narrow width and concentrated data distribution of the confidence interval indicate that the experiment adopts a sufficient sample size, with low dispersion and high stability of the localization error measurement results. Meanwhile, the mean value of 1.57 pixels falls entirely within the confidence interval, which demonstrates that the average error of the proposed Hough transform localization algorithm is statistically credible. The experimental results are free from accidental deviations and can stably and objectively reflect the actual localization accuracy of the algorithm.

### 3.2. Classification performance evaluation of front/back pose recognition

**Fig 9:**
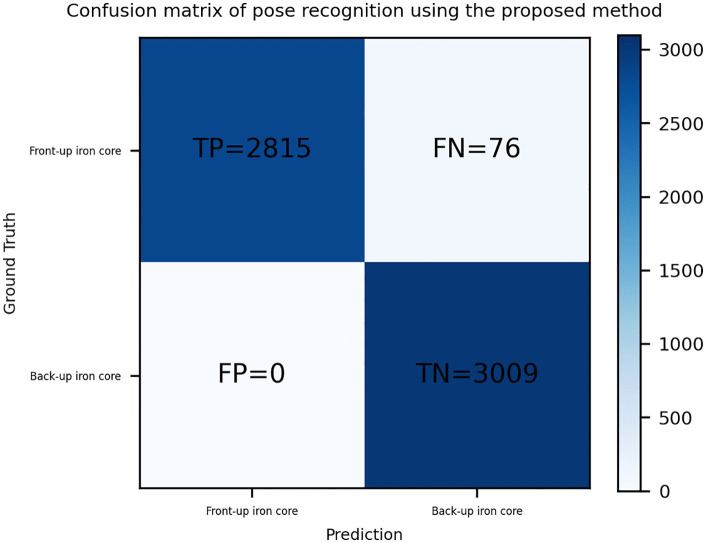
Confusion matrix of pose recognition using the proposed method.

Considering that only front-up iron cores can be grasped by the robotic manipulator, the recognition task belongs to a high-precision industrial scenario, and missed detections of front-up cores are tolerable, whereas false detections are unacceptable. Thus, for front-up iron cores, the detection strategy follows the principle that missing detection is preferable to false detection. To further quantitatively evaluate the classification performance of the iron core front/back pose recognition algorithm and intuitively analyze the false detection and missing detection cases of the model, a binary confusion matrix is constructed to compensate for the limitation that a single accuracy index cannot reflect the detailed classification defects of the model. In this experiment, a total of 5900 buzzer iron core samples, including 2891 front-up iron cores and 3009 back-up iron cores, are adopted as test data. The sufficient sample size and comprehensive working condition coverage can objectively and truly reflect the practical recognition performance of the algorithm in industrial scenarios. Taking front-side iron cores as positive samples and back-side iron cores as negative samples, this study systematically counts the correspondence between the ground truth categories and predicted categories of all test samples to accurately distinguish various classification errors. Based on the experimental results of the 5900 iron core test samples, the confusion matrix of pose recognition is shown in Fig 9.

It can be observed from the confusion matrix that the proposed algorithm produces no false positive (FP) samples. No back-up iron cores are misclassified as front-up samples, which brings a zero false detection rate and a recognition precision of 100% for front-up iron cores. This completely avoids industrial risks such as robotic manipulator grasping failure caused by unqualified iron cores. Only 64 front-up iron cores are mistakenly identified as back-side samples, corresponding to minor false negative (FN) errors, which leads to a small number of missed detections of front-up iron cores. This result conforms to the industrial detection principle of “prioritizing missed detection over false detection.” The classification performance is evaluated by the front/back recognition accuracy, given by Equation (5):


$$\begin{array}{c} \text{Accuracy}=\frac{\text{TP}+\text{TN}}{\text{TP}+\text{TN}+\text{FP}+\text{FN}} \end{array}$$ (5)

where TP, TN, FP, and FN denote true positives, true negatives, false positives, and false negatives, respectively. Using this formula, the front/back recognition accuracy for the cores is calculated to be 98.71%, further demonstrating the reliability of the experimental data. The confusion matrix analysis fully verifies that the proposed front-back recognition algorithm based on central edge pixel statistics possesses extremely low classification errors and stable performance, which can satisfy the high-precision industrial detection requirements for automatic grasping of buzzer iron cores.

### 3.3. Notch-angle measurement error analysis

**Fig 10:**
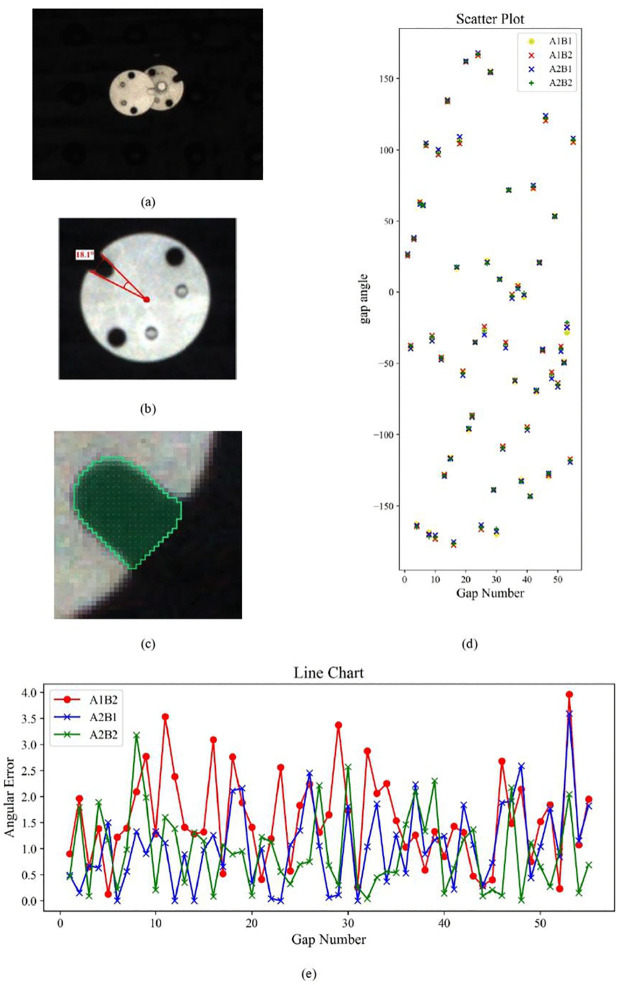
Notch-angle measurement error analysis experimental images. (a) Extreme working case (b) Angle diagram at the core notch (c) Pixel-level annotation visualization at the notch (d) Scatter plot of the four sets of notch-angle data (e) Line chart of absolute errors for the three sets of notch-angle data.

Before evaluating the accuracy of notch angle measurement, the detection quality of the notches themselves must first be assessed. A total of 2891 front-up iron cores’ notches were tested, among which 2887 were successfully detected with only four missed detections. These rare missed cases correspond to the extreme working case shown in Fig 10(a): Since notch detection and angle calculation both rely on binary images, when two iron cores overlap and the notch of the upper workpiece directly faces the main body of the lower iron core, the binarization process converts the upper notch area into white pixels with a grayscale value of 255. This area should have been black pixels with a grayscale value of 0, so it blends seamlessly with the lower core body and ultimately leads to missed notch detection. These four missed detections are rare extreme cases that barely affect overall detection performance. With reliable notch detection verified, the study further quantifies notch angle measurement accuracy and analyze its localization-induced errors.

In actual production, every core must be placed at a fixed orientation so that its notch faces the same direction. Hence, the measurement error of the notch angle is crucial for correct core placement. The main influencing factors are notch position and core center localization errors. Fig 10(b) illustrates the opening angle of the arc at the core notch. The core center used is the geometric centroid obtained from the pixel-level annotations described in Section 3.1, and the arc angle is measured using the engineering dimension tool in Microsoft Visio. Measuring 50 cores yields an average arc angle of approximately 18.1°, implying that an angular error below 9.05° (half of 18.1°) is acceptable for the proposed algorithm. To analyze the notch-angle measurement error and its main influencing factors, 200 front-up cores are prepared. The notch of each core in the images is annotated at the pixel level using the image-labeling software Supervise.ly. The resulting pixel-level annotation visualization is shown in Fig 10(c). The geometric centroid of each annotated region is taken as the ground-truth notch localization for that core. The core center is set to the manually annotated ground-truth centroid (A1) or the center detected by the Hough-circle detector (A2), while the notch localization is set to the manually annotated ground-truth notch centroid (B1) or the notch coordinates obtained by the circular-traversing method proposed in this paper (B2). These factors are crossed to produce four combinations—A1B1, A1B2, A2B1 and A2B2—yielding four sets of notch angles. The scatter plot in Fig 10(d) presents the notch-angle measurements for a subset of iron cores under the four combinations. The four data sets for the same notch are nearly identical, with only minor deviations. To obtain an accurate analysis of notch-angle measurement error, the A1B1 result is taken as the reference (standard) angle, and the absolute angular error of each of the other three groups relative to this reference is calculated. The three resulting error sets are plotted in Fig 10(e).

The line plot shows that the A1B2 group exhibits the largest mean error (1.75°), which remains below the 9.05° tolerance, thus confirming sufficiently accurate notch localization. The A2B1 error is smaller than that of A1B2, indicating that imprecise notch localization contributes more to the notch-angle measurement error than imprecise core center localization. In other words, the dominant factor in angle measurement error is the localization error of the notch. The mean errors of A2B2 and A2B1 are 1.06° and 1.08°, respectively, which are nearly identical, with A2B2 being slightly smaller. This implies that when A2B2 is used, both core center (A2) and notch position (B2) localizations suffer similar deviations caused by lens distortion, so the resulting value actually approaches the true notch angle (A1B1) more closely. The proposed notch-angle measurement algorithm (A2B2) exhibits a mean error of only 1.06° with a variance of 1.225, a level that does not affect buzzer winding and meets production requirements.

To quantify the statistical stability and result reliability of angle measurement errors, a 95% confidence interval analysis is conducted on 100 sets of notch angle error data of iron cores. It can be evaluated according to Equation (4) presented in Experiment 3.1. Using this formula, the calculated 95% confidence interval of the angle measurement error for the A2B2 combination of the proposed algorithm is [0.83°, 1.29°]. The confidence interval presents a narrow range and low fluctuation, with the average error of 1.06° located at the center of the interval, which indicates excellent repeatability and high statistical confidence of the angle measurement experimental data. Furthermore, the upper bound of the confidence interval is far lower than the industrial error threshold of 9.05°. The statistical results further verify that the proposed notch angle detection algorithm features controllable error and stable performance, which fully meets the precision requirements for industrial winding production of buzzer iron cores.

### 3.4. Comparison with YOLO algorithms

**Fig 11:**
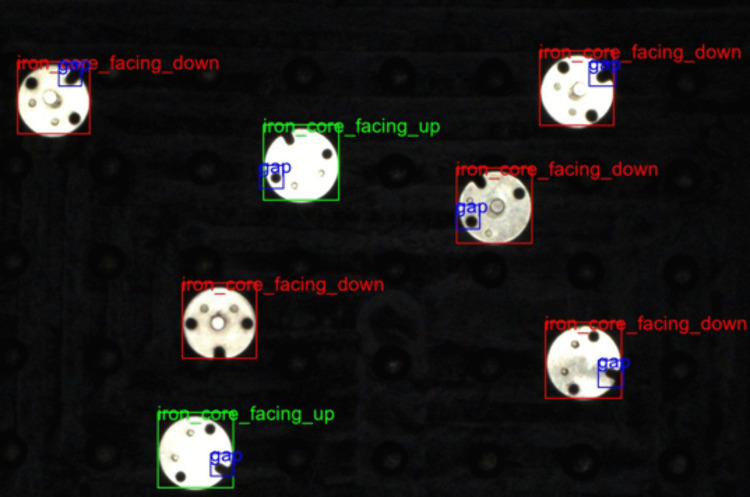
Test result image.

**Fig 12:**
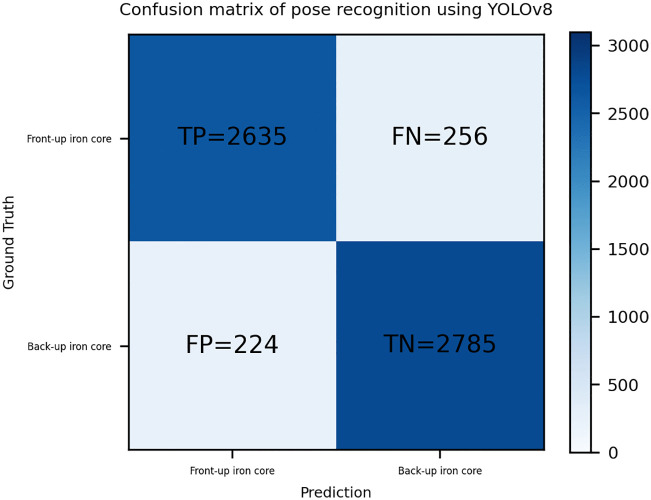
Confusion matrix of pose recognition using YOLOv8.

YOLO is a widely used object detection algorithm that has been extensively applied across various fields such as industry, transportation, agriculture, and security. Among all models in the YOLO series, YOLOv8 represents the latest and most popular version for industrial applications. Accordingly, we adopt YOLOv8 as the deep learning model for comparative experiments in this paper. Since buzzer iron core detection involves a multi-task system integrating localization, pose recognition, and angle measurement, it is difficult for YOLO to simultaneously and directly output the center coordinates and notch angle of cores facing upright in an end-to-end manner. Accordingly, the experiments employ YOLOv8 as the detection model, labeling three object classes—front-up cores, back-up cores, and the notches of all cores—and then used these annotations to calculate each core's center coordinates and notch angle. The detailed steps are as follows:

1) Image Annotation: A total of 1,000 core images were collected. With the image annotation tool LebelImg, holistic annotations were performed on all non-overlapping front-up cores, covering their notches and holes. Since the notch geometry is identical for both front-up and back-up cores, notches of two core orientations were annotated to facilitate model training. The final annotated dataset contains 9,532 front-up cores, 10,109 back-up cores, and 19,641 notches in total.
2) Model modification: In the 3072 × 2048 core images, the notch width is only about 7 pixels. However, the standard YOLOv8 model requires a 640 × 640 input, which is too small to preserve notch detail. Consequently, the input resolution was raised to 1920 × 1920.
3) Model training: The hardware environment for training and inference is listed in Table 1. Raising the input resolution substantially increases both computational load and memory consumption, so the mini-batch size is set to 16. The other hyper-parameters are fixed at 400 epochs, an initial learning rate (lr0) of 0.01, and 8 data-loading workers.
4) Testing the trained model: Given the need for high precision in a densely packed scenario, the confidence threshold is set to 0.5 to suppress false positives and the IoU threshold to 0.4 to eliminate duplicate boxes. Two hundred images were acquired and evaluated using the image-acquisition module developed in this study. Representative YOLOv8 evaluation results are shown in Fig 11, where green bounding boxes indicate front-up cores, red bounding boxes indicate back-up cores, and blue bounding boxes indicate notches. The figure reveals that YOLOv8 successfully localizes every core in the images. Recognition of front-up cores is generally accurate, with only minor missed and false detections. However, notch detection performs poorly, exhibiting numerous omissions and false positives.

5) Core localization: Based on the detection results for front-up and back-up cores, the geometric center of each localized core region is computed, and its Euclidean distance to the corresponding ground-truth center is determined. Following the procedure described in Section 3.1, the average relative localization error is 6.83%, equivalent to a localization accuracy of 94.17%.
6) Front/back classification: Cores predicted as “front-up” are treated as positive samples and those predicted as “back-up” as negative samples. The classification performance can be evaluated according to Equation (5) presented in Experiment 3.2. Using this formula, the front/back classification accuracy for the cores is calculated to be 91.86%. The confusion matrix for pose recognition based on YOLOv8 is shown in Fig 12.

7) Notch-angle measurement: Before assessing the accuracy of notch-angle measurement, the quality of notch detection itself must be evaluated. Notches are treated as positive samples, whereas core holes and any other detected objects are treated as negatives. The detection performance is quantified by the recall and precision of notch detection, given by Equation (6) and Equation (7):

$$\begin{array}{c} \text{Recall}=\frac{\text{TP}}{\text{TP}+\text{FN}} \end{array}$$ (6)

$$\begin{array}{c} \text{Precision}=\frac{\text{TP}}{\text{TP}+\text{FP}} \end{array}$$ (7)

Using the above formulas, the notch-detection recall and precision are found to be 52.68% and 75.13%, respectively. Next, the geometric center of each true-positive notch is calculated and its opening angle is derived. Comparison with the ground-truth angles acquired via the A1B1 combination described in Section 3.3 yields an average angular error of approximately 8.67° for the YOLOv8 model.

The performance metrics of YOLOv8 and the proposed method are shown in Table 2:

**Table 2:** Performance metrics.

Method category	Localization accuracy	Front/back classification accuracy	Notch detection recall	Notch detection precision	Notch-angle Error	Inference speed
YOLOv8	94.17%	91.86%	52.68%	75.13%	8.67°	207ms
Proposed method	97.39%	98.71%	99.86%	100%	1.06°	186ms

Overall, for the detection task specified in this paper, the proposed method surpasses YOLOv8 across all performance metrics. For core localization and front/back classification, the difference between YOLOv8 and our approach is marginal. However, in the angle-measurement module, a noticeable gap exists between the two methods. YOLOv8 suffers from certain false detections and missed detections for notch targets. Finally, in terms of practical deployment and computational cost, the proposed method achieves higher inference speed, offering a clear advantage in buzzer iron-core detection tasks.

## 4. Discussion

This study proposes a machine vision-based detection method integrating target localization, pose recognition and notch-angle measurement for buzzer iron core industrial feeding scenarios. By combining optimized image identification techniques and Hough transform algorithm, the proposed system is successfully deployed on automated iron core feeding equipment, achieving accurate and efficient intelligent grasping and handling of buzzer iron cores. A comprehensive comparison with the mainstream YOLOv8 detection model and systematic industrial field verification further demonstrate the superiority and engineering practicability of the proposed method.

In terms of detection performance and algorithm accuracy, the proposed method exhibits comprehensive advantages over YOLOv8 in buzzer iron core detection tasks. The method proposed in this paper achieves slightly better performance than YOLOv8 in iron core localization and front/back pose recognition. Specifically, its localization accuracy is 3.22% higher, and front/back classification accuracy is improved by 6.85% compared with YOLOv8. Moreover, the proposed approach achieves markedly superior performance in the critical task of notch angle measurement. YOLOv8 imposes strict input resolution constraints, so the input image has to be downscaled. Once the image resolution is reduced, its detection performance for tiny targets will degrade drastically, and the notches on iron cores are exactly such small objects. Furthermore, the YOLOv8 network continuously downsamples feature maps during forward inference, which blurs the features of notches. This leads to a substantial decline in detection accuracy, accompanied by frequent false detections of holes as notches and missed detections of real notches. Among the notches that YOLOv8 does succeed in detecting, the mean angular error is about 8.67°, nearly approaching the industrial tolerance of 9.05°. In contrast, our method achieves a 2.61% localization error, a 1.06° angle error and 100% front-side recognition precision, with lower computational cost and better real-time performance for industrial feeding tasks.

The comparison of inference speeds fully demonstrates the remarkable superiority of the method proposed in this paper. YOLOv8 consumes approximately 207ms for single-image inference, while our method achieves a moderate speed improvement with an inference time of around 186ms. Notably, the proposed algorithm processes full high-resolution input images, whereas YOLOv8 downsamples input images at the model input stage. This contrast further verifies the speed advantage of our algorithm, which delivers real-time computational performance suitable for online industrial detection.

Apart from algorithm performance superiority, the industrial applicability, operational stability and practical production capacity of the proposed system are fully verified through actual on-site debugging and long-term endurance tests on the buzzer iron core feeding production line. The proposed algorithm was fully validated via on-site joint debugging with a parallel industrial robotic manipulator. It implements complete operational procedures, including image acquisition, core localization, pose recognition, notch angle calculation, and real-time data transmission for robotic grasping adjustment. The algorithm's absolute localization error of ±0.109 mm is well within the 1.2 mm gripper tolerance, guaranteeing reliable grasping. Batch tests on over 12,000 workpieces yielded a high grasping success rate of 99.94%, with failures only occurring under extreme working conditions where iron cores overlap with their notches facing the lower part of the iron cores, and this issue can be resolved by optimizing feeding parameters. A 72-hour continuous endurance test confirmed stable system operation without crashes, frame loss or parameter drift, requiring only routine maintenance. The system achieves a minimum single-cycle processing time of 0.68 s and a stable throughput of 87 parts per minute, fully satisfying the industrial assembly line's real-time and mass production requirements.

In summary, the proposed method outperforms YOLOv8 in detection accuracy, angle measurement precision and real-time performance for buzzer iron core detection. Validated through comprehensive industrial tests, it exhibits reliable grasping performance, high operational stability and favorable production efficiency, possessing great practical value and promising industrial application prospects in automated iron core feeding and assembly.

## 5. Conclusion

A machine vision-based detection method for buzzer iron cores that integrates localization, pose recognition, and angle measurement. By combining machine vision with image identification techniques, the system can be deployed on automated iron core feeding equipment. Experiments show that the adopted Hough transform algorithm achieves an average relative localization error of only 2.61%, delivers 100% recognition precision for front-up iron cores, and measures the notch angle with a mean error of 1.06°. Compared with YOLOv8, the approach offers higher detection accuracy while satisfying the real-time requirements of industrial feeding tasks.
